# Circadian activity of small brown bear populations living in human-dominated landscapes

**DOI:** 10.1038/s41598-022-20163-1

**Published:** 2022-09-22

**Authors:** Aurora Donatelli, Gianluca Mastrantonio, Paolo Ciucci

**Affiliations:** 1 Department of Biology and Biotechnologies “Charles Darwin”, Sapienza University of Rome, Rome, Italy; 2 Department of Mathematics (DISMA), Politecnico di Torino, Torino, Italy

**Keywords:** Animal behaviour, Zoology

## Abstract

Whereas numerous studies on large carnivores have focused on analyzing spatial patterns and habitat use, the temporal dimension of their activity has been relatively little investigated, making this a topic of growing interest, especially in human-dominated landscapes. Relict and isolated Apennine brown bears (*Ursus arctos marsicanus*) have been living in a human-modified landscape since millennia, but no information is available on their activity patterns. By means of GPS telemetry (26,880 GPS locations collected from 18 adult Apennine brown bears) we investigated their circadian rhythms, using hourly movement rates as an index of bear activity. Based on a Bayesian modeling approach, circadian activity of Apennine brown bears was described by a bimodal curve, with peaks of activity around sunrise and sunset. We revealed seasonal effects, with bears exhibiting higher movement rates throughout the mating season, but no relevant influence of sex. In addition, bears increased their movement rate at distances < 100–500 m to roads and settlements exclusively during spring and late summer, suggesting a trade-off between foraging opportunities and risk avoidance. The absence of a marked nocturnality in Apennine brown bears suggests a relatively low degree of habitat encroachment and disturbance by humans. Yet, the occurrence of crepuscular activity patterns and the responses in proximity of anthropogenic landscape features likely indicate a coadaptation by bears to human presence through a shift in their temporal niche. Further studies should aim to unveil fitness implications of such modifications in activity patterns.

## Introduction

For wildlife species, survival and reproductive instincts can be fundamental factors determining animals’ activity, inducing individuals to temporally segregate from predators or overlap with prey^1^, and to coordinate for mating^2,3^. Circadian rhythms in animals, defined as cyclicity in behavior, physiology, hormone levels and biochemistry on a daily basis^4^, are an adaptation to the changing environmental conditions that characterize the 24 hours^4^. In mammals, a number of genes are responsible for the expression of circadian rhythmicity^5^, which is controlled by a master pacemaker made of two clusters of neurons in the suprachiasmatic nucleus (SCN) of the hypothalamus^4,5^. The central oscillator in the SCN generates an endogenous rhythmicity, following a cycle of about 24 hours, which can be entrained and modified by the interaction between physiological and external factors (‘Zeitgeber’)^6,7^, such as temperature^8^ and light^7^. This circadian clock regulates a variety of processes and physiological parameters, including wakefulness and rest, metabolism and blood pressure^5^.

Among external factors, human disturbance and encroachment may profoundly impact the activity rhythms of wildlife species, clearly depending on the nature and extent of human pressure^9^. Humans are perceived as a predator to which animals associate a risk and whose perception differs among species, individuals, seasons, and times of the day, varying according to the type of anthropogenic activity^9,10^. In such circumstances, especially in human-modified landscapes, wildlife species can increase nocturnal activity, hence adapting their spatio-temporal niche, with the aim to avoid contact with humans^9^. While this flexibility in wildlife’s circadian rhythms is a key component of their adaptive strategies for co-habiting with humans^11^, it is also likely that drastic changes in activity occur at the expense of the individual's fitness, causing stress, reducing foraging efficiency and reproductive success, eventually with cascading effects across entire ecological communities^12,13^.

Large carnivores, in particular, are highly sensitive to human activity^14,15^, and they represent model species to study the nature and extent of wildlife’s behavioral adaptation to human-modified landscapes^16^. Mountain lions (*Puma concolor*) in developed areas of California, for instance, are more active at night than during the day, and they move further and expend more calories near human settlements at night compared to areas with lower human development^17^. Wolves (*Canis lupus*) living in human-dominated landscapes of Europe, have adapted to high densities of humans and infrastructures by displaying habitat-mediated spatio-temporal behavioral adaptations, including nocturnal activity, to reduce the risk associated with encountering humans^18–20^.

Being widely distributed in varying landscapes and across ecological conditions^21^, brown bears (*Ursus arctos*) exhibit a variety of activity patterns^22,23^ and represent a good model species to investigate the anthropogenic effects on wildlife’s circadian rhythms. In North America, for instance, brown bears living in more pristine conditions and subject to lower human disturbance tend to be less crepuscular and nocturnal (i.e., active around sunset and sunrise, and during an interval between an arbitrary time shortly after sunset and before sunrise, respectively) compared to populations living in the highly anthropized Europe^24^. On the other hand, brown bears’ circadian rhythms in North America are influenced by a number of factors, including anthropogenic pressure^25^, seasonal shifts in diet^23,26^, and sex^25^. For instance, while bears’ efficiency in catching elk calves is higher during the night in spring and summer, foraging on fruits is facilitated during daylight in the hyperphagic period^23,26^. Grizzly bears (*Ursus arctos horribilis*) in the Grand Teton National Park become more nocturnal when near roads and developed sites, although females were found to be more day-active than males, possibly to reduce the risk of conflict with adult males^25^. Bears in Europe, instead, have been more consistently reported to exhibit a bimodal crepuscular circadian pattern, with varying levels of nocturnal activity (e.g., Serbia:^27^, northern Italy:^28^, Slovenia and Croatia:^29^, Pyrenees:^30^). Although a longer history of persecution might have had evolutionary effects on the circadian activity of European bears^31,32^, variation in their daily rhythms has also been associated with the intensity of human disturbance^33^. Bears in Sweden, for instance, quickly respond to human-derived risks by becoming more nocturnal as the hunting season starts^34^. The presence of roads, which through enhanced human access increases disturbance and risk of mortality, is also known to affect the activity rhythms of bears^31^. Accordingly, in Sweden, bears living in areas of high road density were more active at night compared to those living in roadless regions^22^.

With the exception of Scandinavia, where a number of studies on bears’ activity have been recently carried out (e.g.,^22,35,36^), this topic has been scarcely investigated in other human-modified landscapes of Europe. This is particularly true for brown bear populations living in south-western Europe, comprising few, small, and isolated populations that face high risks of extinction by living in close association with humans^37,38^. In such conditions, understanding how bears shape their activity in response to humans is paramount to plan and assess their long-term conservation.

The Apennine brown bear (*Ursus arctos marsicanus*) is an autochthonous, relict, and isolated population living in the central Apennines of Italy^39^. Separated from the Alpine population probably well before 1000 years ago^40^, Apennine bears currently number about 50 individuals^41^ and are critically endangered under both local and European IUCN criteria^38,42^. Due to their prolonged isolation, Apennine bears feature a very low genetic variation and a high degree of phenotypic differentiation^40^, representing a unique conservation unit. They are generally active from mid-March to mid-December (P. Ciucci, unpubl. data) and have a mostly vegetarian diet, with slight differences between sexes^43,44^. Although several ecological and conservation-related aspects of this imperiled bear population have been investigated during the past decade, knowledge on their activity rhythms is currently lacking. Because Apennine brown bears have been living alongside humans since millennia^40^ and have never been extirpated nor augmented with founders from other European populations, they represent an ideal case study to investigate how their temporal niche has been shaped by millennia of coevolution with humans.

In this study, we used Global Positioning System (GPS) telemetry to investigate circadian activity rhythms of Apennine bears residing in the Abruzzo, Lazio and Molise National Park (PNALM), central Italy. Using hourly movement rates as an index of activity, we developed Bayesian models to investigate the relationship between activity and time of day, accounting for sex and season, as well as distance from roads and settlements. Specifically, due to the long history of persecution, their isolated distribution, and the multi-use landscape in which they live, we hypothesized that Apennine bears exhibit a marked nocturnality as to minimize encounter rates with humans (H1). We further hypothesized that movement rates differ between males and females (H2), and seasonally (H3), the latter leading to two predictions: bears show higher movement rates throughout their active bouts during the mating period in early summer, when they consistently roam to search for a partner (P1), and bears increase their nocturnal movements during early and late summer, both to avoid increased human disturbance (e.g., tourism, hiking, livestock grazing), and as a thermoregulatory response to higher temperatures (P2). We also hypothesized that the movement rate of individual bears is influenced by the distance to roads and/or settlements (H4), leading us to predict that bears increase their movement rate in proximity of roads and settlements, where they might have a higher perception of risk (P3).

## Methods

### Study area

We conducted our study on Apennine brown bears residing in the PNALM, located in the central Apennines, Italy, including the buffer area surrounding the park. The 2500 km^2^ study area is mainly mountainous, ranging from 400 to 2285 m, with average temperatures ranging from 2 °C in January to 20 °C in July^43^. The area is characterized by dry summers and cold winters, with snow cover generally present from mid-December to March. About 60% of the area is covered by deciduous forests, 21.8% by grassland, and 3.5% by agricultural fields. The majority of forests are composed of beech (*Fagus sylvatica*) above 1000 m, and of mixed oak (*Quercus cerris, Q. pubescens*) and fruit trees (*Pyrus pyraster*, *Malus sylvestris*, *Prunus spp.*) at lower elevations^43^. Shrubs include multiple fruiting plants (*Rhamnus alpinus*, *Ribes* spp., *Rosa* spp., *Rubus idaeus* e *Viburnum* spp.).

Two large carnivore species are present in the study area, Apennine bears, with a density of 39.7 bears/1000 km^2^^41^, and wolves, with a density of > 5 wolves/100 km^2^^45^. Large ungulates include red deer (*Cervus elaphus*), roe deer (*Capreolus capreolus*) and wild boar (*Sus scrofa*). Mean road density is 1.1 km/km^2^, while human density is estimated to be around 14.6 inhabitants/km^2^^41^. Both inside the PNALM and in its buffer zone, a number of human activities are allowed, such as tourism, regulated forestry practices and livestock husbandry^39^. Further details on the study area can be found in^41,43^ and^45^.

### Bear data

We used GPS data from 18 adult (≥ 4 years old^46^) Apennine bears, 11 females and 7 males, collected from 2005 to 2010 during their active period, roughly from the end of March to mid-December^47^. The sample of GPS-collared bears included in this work does not include habituated, food-conditioned or otherwise management bears. While the original dataset comprised 29,796 GPS locations, for the scope of the analysis we subsampled 26,880 of them in order to both standardize the acquisition rate (i.e., 1 location/h) across all individuals, and screen the data for outliers^48^. The observed acquisition rate ranged in 79–97%, and mean location error (± SD), estimated through 100 stationary locations, was 24.7 ± 16.3 m.

### Model development

Following^22^, we adopted a Bayesian framework^49,50^ to test our hypotheses. We used R (Version 4.2.0,^51^) and JAGS (Version 4.3.0,^52^) to develop Bayesian models, and in particular the *rjags* and *runjags* packages to communicate from R to JAGS. We used scripts available in^50^ to run Monte Carlo Markov chains (MCMC) and evaluate the chains’ convergence. We preferred a Bayesian approach over a frequentist one (e.g.,^27^), as the former allows for more complex models and a higher precision, especially as the complexity of the models increases^53^. The posterior distribution contains all the information necessary to allow inference without the need for asymptotic theory, resampling methods, and other assumptions often used in frequentist approaches to estimate parameters in complex models^50,53^.

### Model variables

As a response variable in our models we used a proxy of bear activity, that is the Euclidean distance (km) between successive GPS locations collected at hourly intervals (hourly movement rate;^22^). This was square root transformed as the resulting models were more stable and produced a better fit.

Several discrete parameters were included in the models to reflect our hypotheses, namely time of day, sex, and season. Since our interest was to model the activity of bears in each hour of the day, the time of the day parameter (λ_i_) was modeled as a multivariate normal distribution with 24 levels (i = 1, …, 24; see below). To account for seasonality, we made reference to the four dietary seasons of Apennine bears as defined in^44^: spring (March–May); early summer (June–July); late summer (August–September); fall (October–mid-December). We took into account the individual bear variability by incorporating a random effect of the individual in all models. Moreover, in a Geographic Information System (GIS) environment, we calculated the distance (km) from every bear location to primary roads (asphalted of high usage), secondary roads (asphalted of low usage or dirt roads), and human settlements, and included these as continuous variables using an exponential function to realistically represent a decreasing effect at increasing distances^54^.

### Implemented models

We implemented 13 models of growing complexity, testing in each different interaction terms (Table 1). In its general formulation, each model can be written as:

1 $${\text{y}}_{{{\text{tk}}}} = \, \lambda^{*}_{{{\text{tk}}}} + \, \varepsilon_{{{\text{tk}}}}$$

where y_tk_ is the square root transformed response variable (i.e., hourly movement rate), with subscript t representing the date and time of each GPS location, and k = 1, …, 18 the individual indicator. λ^*^_tk_ is the sum of the parameters included in the model and may take on different forms in each model (Table 1). The noise term, ε_tk_, is distributed according to the normal function N(0, σ^2^), where 0 is the expected value and σ^2^ represents the variance.

**Table 1:** Model selection to assess the circadian activity of Apennine bears in the Abruzzo, Lazio and Molise National Park (central Italy, 2005 − 2010).

Model no.	Model formula	Model selection		
		K	DIC	∆DIC
(9)	$$\begin{aligned} \lambda^{*}_{tk} & = \, \lambda_{irs} + \, \omega_{k} + \, \xi_{rs} + \, \beta_{2r} exp\left( { - \varphi_{2r \, } \cdot x_{2tk} } \right) \, \\ & \;\; + \, \beta_{3r} exp\left( { - \varphi_{3r \, } \cdot x_{3tk} } \right) \, + \, \beta_{4r} exp\left( { - \varphi_{4r} \cdot x_{4tk} } \right) \\ \end{aligned}$$	5	12,898	–
(4)	$$\begin{aligned} \lambda^{*}_{tk} & = \, \lambda_{irs} + \, \omega_{k} + \, \mu_{s} + \, \gamma_{r} + \, \beta_{1r} exp\left( { - \varphi_{1r \, } \cdot x_{1tk} } \right) \\ & \;\;\; + \, \beta_{4r} exp\left( { - \varphi_{4r} \cdot x_{4tk} } \right) \\ \end{aligned}$$	3	12,904	6
(5)	$$\begin{aligned} \lambda^{*}_{tk} & = \, \lambda_{irs} + \, \omega_{k} + \, \mu_{s} + \, \gamma_{r} + \, \beta_{2r} exp\left( { - \varphi_{2r} \cdot x_{2tk} } \right) \\ & \;\; \, + \, \beta_{3r} exp\left( { - \varphi_{3r} \cdot x_{3tk} } \right) \, + \, \beta_{4r} exp\left( { - \varphi_{4r} \cdot x_{4tk} } \right) \\ \end{aligned}$$	4	12,906	8
(8)	$$\begin{aligned} \lambda^{*}_{tk} & = \, \lambda_{irs} + \, \omega_{k} + \, \xi_{rs} + \, \beta_{1r} exp\left( { - \varphi_{1r} \cdot x_{1tk} } \right) \\ & \;\; + \, \beta_{4r} exp\left( { - \varphi_{4r} \cdot x_{4tk} } \right) \\ \end{aligned}$$	4	12,913	15
(6)	$$\begin{aligned} \lambda^{*}_{tk} & = \, \lambda_{irs} + \, \omega_{k} + \, \xi_{rs} + \, \beta_{1} exp\left( { - \varphi_{1 \, } \cdot x_{1tk} } \right) \\ & \;\;\; + \, \beta_{4} exp\left( { - \varphi_{4} \cdot x_{4tk} } \right) \\ \end{aligned}$$	2	12,964	66
(7)	$$\begin{aligned} \lambda^{*}_{tk} & = \, \lambda_{irs} + \, \omega_{k} + \, \xi_{rs} + \, \beta_{2} exp\left( { - \varphi_{2} \cdot x_{2tk} } \right) \\ & \;\; + \, \beta_{3} exp\left( { - \varphi_{3 \, } \cdot x_{3tk} } \right) \, + \, \beta_{4} exp\left( { - \varphi_{4 \, } \cdot x_{4tk} } \right) \\ \end{aligned}$$	2	12,984	86
(12)	$$\begin{aligned} \lambda^{*}_{tk} & = \, \lambda_{ir} + \, \omega_{k} + \, \gamma_{r} + \, \beta_{1r} exp\left( { - \varphi_{1r} \cdot x_{1tk} } \right) \\ \, & \;\; + \, \beta_{4r} exp\left( { - \varphi_{4r} \cdot x_{4tk} } \right) \\ \end{aligned}$$	3	13,249	351
(13)	$$\begin{aligned} \lambda^{*}_{tk} & = \, \lambda_{ir} + \, \omega_{k} + \, \gamma_{r} + \, \beta_{2r} exp\left( { - \varphi_{2r} \cdot x_{2tk} } \right) \\ & \;\; + \, \beta_{3r} exp\left( { - \varphi_{3r} \cdot x_{3tk} } \right) \, + \, \beta_{4r} exp\left( { - \varphi_{4r} \cdot x_{4tk} } \right) \\ \end{aligned}$$	4	13,268	370
(10)	$$\begin{aligned} \lambda^{*}_{tk} & = \, \lambda_{ir} + \, \omega_{k} + \, \gamma_{r} + \, \beta_{1} exp\left( { - \varphi_{1} \cdot x_{1tk} } \right) \\ & \;\; + \, \beta_{4} exp\left( { - \varphi_{4} \cdot x_{4tk} } \right) \\ \end{aligned}$$	1	13,312	414
(11)	$$\begin{aligned} \lambda^{*}_{tk} & = \, \lambda_{ir} + \, \omega_{k} + \, \gamma_{r} + \, \beta_{2} exp\left( { - \varphi_{2} \cdot x_{2tk} } \right) \\ & \;\; + \, \beta_{3} exp\left( { - \varphi_{3} \cdot x_{3tk} } \right) \, + \, \beta_{4} exp\left( { - \varphi_{4} \cdot x_{4tk} } \right) \\ \end{aligned}$$	1	13,331	433
(2)	$$\begin{aligned} \lambda^{*}_{tk} & = \, \lambda_{i} + \, \omega_{k} + \, \mu_{s} + \, \gamma_{r} + \, \beta_{1} exp\left( { - \varphi_{1} \cdot x_{1tk} } \right) \\ & \;\; \, + \, \beta_{4} exp\left( { - \varphi_{4} \cdot x_{4tk} } \right) \\ \end{aligned}$$	0	13,564	666
(3)	$$\begin{aligned} \lambda^{*}_{tk} & = \, \lambda_{i} + \, \omega_{k} + \, \mu_{s} + \, \gamma_{r} + \, \beta_{2} exp\left( { - \varphi_{2} \cdot x_{2tk} } \right) \\ \, & \;\;\; + \, \beta_{3} exp\left( { - \varphi_{3} \cdot x_{3tk} } \right) \, + \, \beta_{4} exp\left( { - \varphi_{4} \cdot x_{4tk} } \right) \\ \end{aligned}$$	0	13,582	684
(1)	$\lambda^{*}_{tk} = \, \lambda_{i} + \, \omega_{k}$	0	13,763	865

K, number of parameters in interaction; DIC, Deviance Information Criterion score; ∆DIC, DIC score difference with respect to the best selected model. See the text for the symbols of the model parameters.

Specifically, in its most basic form (Table 1: Model 1), λ^*^_tk_ takes the form:

2 $$\lambda^{*}_{{{\text{tk}}}} = \lambda_{{\text{i}}} + \omega_{{\text{k}}}$$

where λ_i_ represents the effect of the time of the day, with subscript i = 1, …, 24 being the time of the day expressed in hours, and ω_k_ accounts for the random effect of the individual k, assumed to come from a normal distribution N(α, ρ^2^), with α the expected value and ρ^2^ the variance. λ_i_ is modeled with the multivariate normal distribution

3 $$\lambda \, \sim {\text{ MN}}\left( {{\mathbf{0}}, \, \Sigma } \right)$$

where λ = (λ_1_, …, λ_24_) and **0** is a vector of zeros of dimension 24. To represent the circular nature of the temporal intervals between hours, we assume that the covariance between elements in λ decreases with increasing circular distance in time across the 24-h period (i.e., the closer two hours, the more similar the movement rate is expected to be^55^). Hence, each element in position (i, i') in the symmetric 24 × 24 covariance matrix Σ, with i, i′ = 1, 2, …, 24 representing the hours of the day, is equal to

4 $${\text{exp}}\left( { - \nu \cdot {\text{min}}\left( {\left| {{\text{i}} - {{{\rm i}^{\prime}}}} \right|,{ 24 } - \, \left|{\text{i}} - {{{\rm i}^{\prime}}}\right|} \right)} \right){/}\psi$$

with min(|i − i′|, 24−|i − i′|) the circular distance in time (e.g., the distance between 24:00 and 01:00 AM is equal to 1). Instead, since^22^ does not formulate the distance between hours taking into consideration the cyclical nature of the 24-h clock (i.e., the hour of the day effect was not defined to have a cyclical nature between hours belonging to consecutive days), their approach can potentially introduce biases and/or increase the uncertainty of the estimates.

The model in Eq. (2) represents our base model, according to which circadian rhythms of bears depend exclusively on endogenous factors. In more complex models (Table 1), the term λ^*^_tk_ also includes the effect of sex (μ_s_), the effect of season (γ_r_), and the interaction between the two (ξ_rs_) as fixed factors. Here, subscript s = 1 is for females and s = 2 is for males, while subscript r = 1 is for spring, r = 2 for early summer, r = 3 for late summer, and r = 4 for fall. We then included the effect of sex and season in interaction with the hour of the day in λ_irs_, or solely the effect of season in interaction with the hour of the day in λ_ir_, to account for a variable effect of these factors throughout the 24 hours. We also considered the effect of the distance from each GPS location to roads (primary + secondary roads: x_1tk_), primary roads only (x_2tk_), secondary roads only (x_3tk_) and settlements (x_4tk_). These distances are modeled as an exponential function with decay parameter φ, that takes on only positive values to ensure that the exponential function reaches an asymptote for larger distances, and a multiplier β (e.g., β_1_ exp(− φ_1 ·_ x_1tk_) for the totality of the road network). Finally, we modeled the anthropogenic effects in interaction with season r (e.g., β_1r_ exp(− φ_1r ·_ x_1tk_) for the totality of the road network), to account for a possible variability in intensity of the disturbance caused by roads and settlements throughout the year.

We use weakly informative priors for all parameters, to allow the data to be the main influence on the posterior distributions. In particular, the parameters in the distribution of the random effect of the individual are defined as α ~ N(0, 1000) and 1/ρ^2^ ~ Ga(1, 1). The priors on ψ and ν of Eq. (4) are Ga(1, 1) and Unif(3/24, 3/1), respectively, so that the practical range of the covariance function, i.e., the smallest distance that gives a negligible correlation, is in the range of 1–24 hours, and it is needed to be able to identify the parameter^56^. The priors on the effects of sex, season and their interaction are N(0, 1000). The variables in the functions describing the anthropogenic effects were defined as follows: β was modeled with the prior N(0, 1000) and φ with a uniform distribution between 3/4 and 3/0.2, while 1/σ^2^ ~ Ga(1, 1).

For descriptive purposes, we chose the hourly movement rate averaged across the 24 hours as a threshold to differentiate between activity and inactivity of bears.

### Model validation

To implement the MCMC algorithm we ran two chains for each model. We set up a burn-in of 50,000 to assure chains convergence, which we visually verified through graphs built with scripts from^50^, and we further ran the chains for 20,000 iterations with a thin of 10, to obtain a sample of 2000 points from the posterior distribution of each parameter. From these, we estimated the mean and the credibility interval (defined by the interval between the 2.5% and 97.5% quantiles of the posterior distribution) of each variable. We evaluated each model through the Deviance Information Criterion (DIC)^57^, as it is of straightforward calculation and it is given as an output by JAGS^52^.

## Results

**Figure 1:**
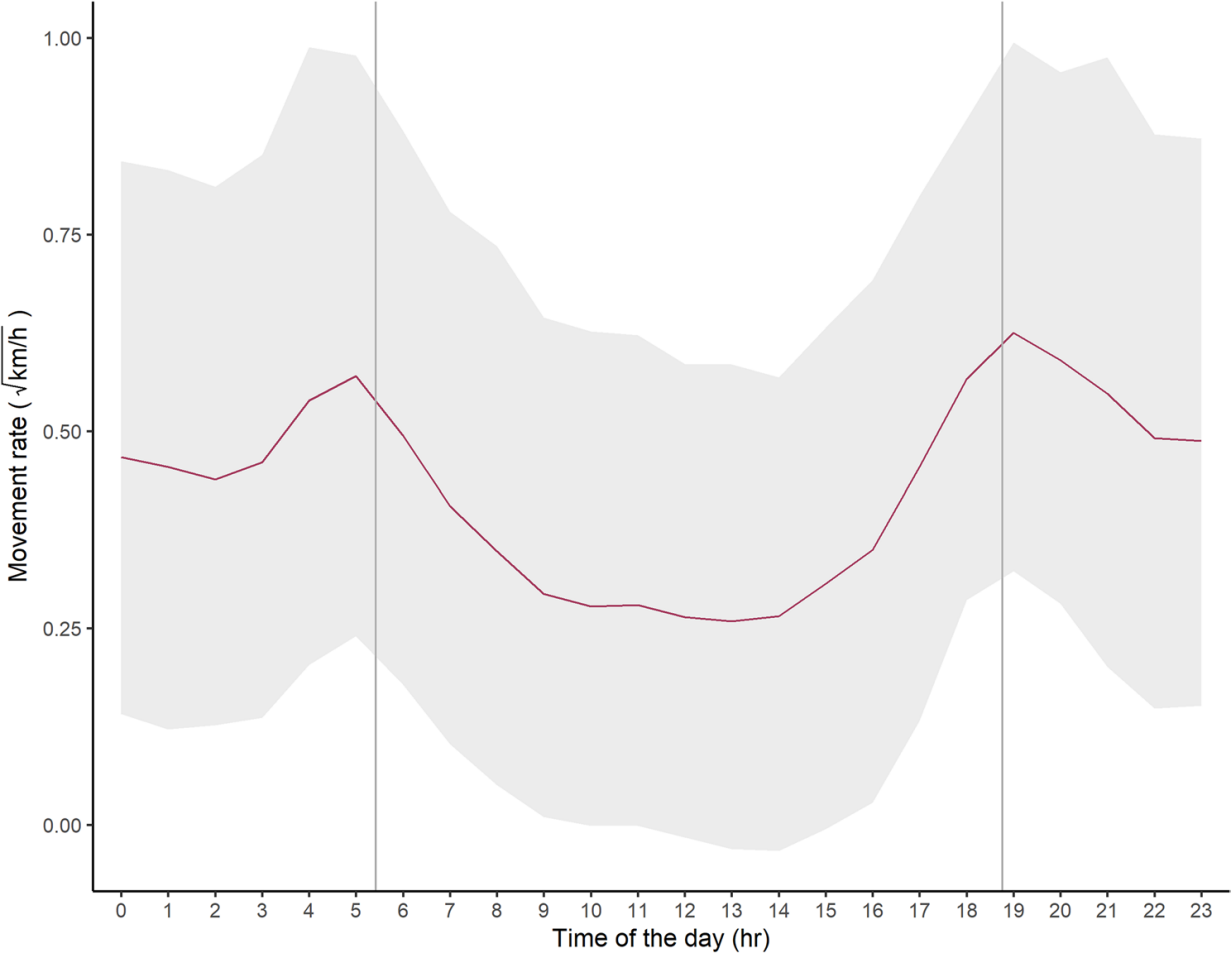
Circadian rhythms of activity of Apennine brown bears indexed by hourly movement rates and based on 26,880 GPS locations recorded at 1-h intervals of 18 adult Apennine bears (11 females, 7 males) in the Abruzzo, Lazio and Molise National Park (central Italy, 2005–2010). Estimated values refer to a bear population with a 50:50 sex ratio, based on the best selected Bayesian model. Grey vertical lines represent the sunrise and sunset times, while the grey area represents the 95% credibility interval.

According to the best selected model (Table 1), Apennine bears showed an overall bimodal, crepuscular distribution of their movement rates (Fig. 1). We revealed two peaks of activity, around 5 AM and 7 PM, during which bears moved on average (± SD) 0.36 ± 0.23 km/h and 0.42 ± 0.22 km/h, respectively (Fig. 1). The overall movement rate across the 24 hours averaged (± SD) 0.22 ± 0.15 km/h, ranging from 0.13 ± 0.11 km/h across the resting periods to 0.30 ± 0.19 km/h throughout the active periods (Table 2).

**Table 2:** Estimated movement rates of adult Apennine brown bears according to the sex and period of the day, and based on the best selected Bayesian model (see Table 1).

Sample	Period	Movement rate (Km/h)	
		Mean (± SD)	Min – Max
Both sexes (n = 18)	24-h period	0.22 ± 0.15	0.20 ± 0.12 (FA) − 0.26 ± 0.16 (ES)
	Active periods (5 PM–1 AM, 3 AM–6 AM)	0.30 ± 0.19	0.27 ± 0.15 (FA) − 0.37 ± 0.21 (ES)
	Inactive periods (7 AM–4 PM, 2 AM)	0.13 ± 0.11	0.11 ± 0.09 (LS) − 0.16 ± 0.14 (SP)
Females (n = 11)	24-h period	0.17 ± 0.10	0.12 ± 0.08 (SP) − 0.19 ± 0.10 (ES)
	Active periods (5 PM–1 AM, 3 AM–6 AM)	0.22 ± 0.12	0.14 ± 0.09 (SP) − 0.25 ± 0.12 (ES)
	Inactive periods (7 AM–4 PM, 2 AM)	0.10 ± 0.07	0.08 ± 0.07 (SP) − 0.11 ± 0.08 (ES)
Males (n = 7)	24-h periods	0.28 ± 0.17	0.22 ± 0.14 (FA) − 0.34 ± 0.18 (SP)
	Active periods (5 PM–1 AM, 3 AM–6 AM)	0.38 ± 0.21	0.29 ± 0.17 (FA) − 0.49 ± 0.22 (ES)
	Inactive periods (7 AM–4 PM, 2 AM)	0.16 ± 0.13	0.12 ± 0.11 (LS) − 0.23 ± 0.15 (SP)

n = sample size (individual bears).

SP, spring; ES, early summer; LS, late summer; FA, fall.

**Figure 2:**
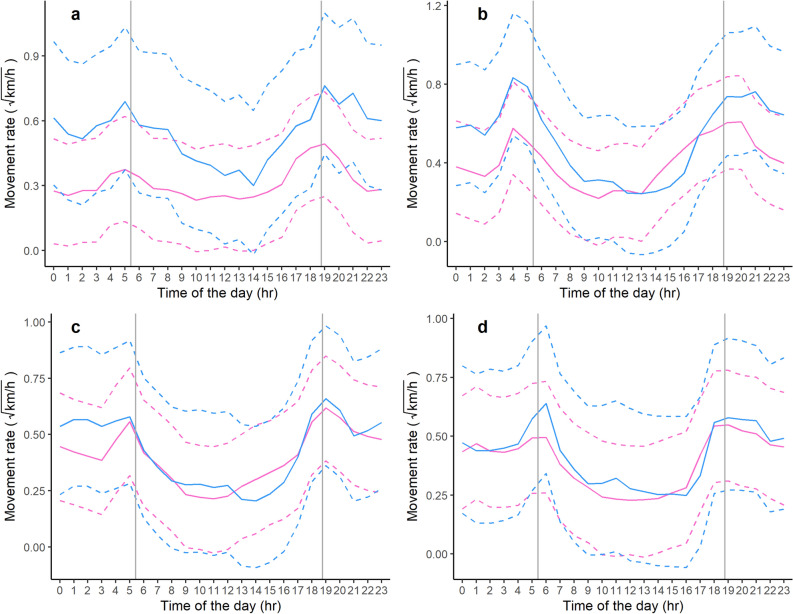
Seasonal circadian rhythms, according to the best selected Bayesian model, of adult male (blue) and female (pink) Apennine brown bears, indexed by hourly movement rates and based on 26,880 GPS locations recorded at 1-h intervals of 18 adult Apennine bears (11 females, 7 males) in the Abruzzo, Lazio and Molise National Park (central Italy, 2005–2010). Grey vertical lines represent the sunrise and sunset times, while dashed lines represent 95% credibility intervals. (**a**) Spring, (**b**) Early Summer, (**c**) Late Summer, (**d**) Fall.

Although sex and season were included in the best selected model as independent parameters in interaction (Table 1), their coefficients were not significant (Table 3). However, their interaction with the hour of the day parameter (λ_irs_) resulted in distinct circadian activity among seasons, but not between females and males (Fig. 2 and Supplementary Fig. S1). Specifically, bears exhibited higher peaks of activity in early summer compared to late summer and fall, and during the first peak of activity (i.e., 3:30–5 AM) in early summer compared to spring (Supplementary Fig. S2). No other differences emerged in circadian patterns between spring, late summer, and fall (Supplementary Fig. S2).

**Table 3:** Coefficients of the best selected Bayesian model to investigate the activity rhythms of Apennine bears resident in the Abruzzo, Lazio and Molise National Park (central Italy, 2005 − 2010).

Variable	Estimated value	95% CIs	
		Lower	Upper
α	0.483	0.256	0.751
σ	0.306	0.304	0.309
ξ_2F_	− 0.198	− 0.631	0.551
ξ_3F_	− 0.013	− 0.491	0.342
ξ_4F_	− 0.350	− 0.773	0.167
ξ_1M_	− 0.092	− 0.436	0.262
ξ_2M_	0.178	− 0.497	0.710
ξ_3M_	0.005	− 0.712	0.478
ξ_4M_	− 0.040	− 0.603	0.511
β_2S_	0.337	0.246	0.436
β_2ES_	0.106	0.024	0.198
β_2LS_	0.084	− 0.117	0.380
β_2F_	0.128	0.033	0.222
β_3S_	0.278	0.130	0.439
β_3ES_	0.131	0.054	0.213
β_3LS_	0.187	0.102	0.287
β_3F_	− 0.042	− 0.082	0.036
β_4S_	0.440	0.048	0.848
β_4ES_	− 0.063	− 0.162	0.029
β_4LS_	0.272	− 0.277	0.805
β_4F_	− 0.174	− 0.258	− 0.090

The reference category for the interaction between sex and season ξ is female in spring, with subscripts being: 1, spring; 2, early summer; 3, late summer; 4, fall; F, female; M, male. The subscripts on the β coefficients of the anthropogenic effects are to be interpreted as: S, spring; ES, early summer; LS, late summer; F, fall; 2, primary roads; 3, secondary roads; 4, human settlements. CIs, credibility intervals.

Movement rate of bears was also affected by proximity to anthropogenic landscape features (Table 3). Specifically, bears increased their movement rate when approaching primary roads at distances < 500 m in spring, although we did not reveal a similar effect in the other seasons (Supplementary Fig. S3). In spring, they also increased their movement rate when < 500 m from secondary roads, while in late summer they only marginally increased their pace when < 100 m from such roads (Supplementary Fig. S4). Similarly, bears increased their movement rate when < 500 m from settlements in spring, and at distances < 100 m in late summer (Supplementary Fig. S5). On average, distances of bear locations to anthropogenic features did not seem to vary throughout the 24 hours (Supplementary Figs. S6–S8); overall, only 4% and 23% of all GPS locations were < 500 m from settlements and roads, respectively.

## Discussion

We explored how season, sex and anthropogenic factors influence circadian rhythms of a relict and isolated bear population living at close quarters with humans since historical times. As such, the Apennine bear population represents a model case study to investigate how human encroachment and disturbance may influence large carnivores’ activity and the extent of bears’ adaptability. Contrary to our H1, we detected a markedly clear bimodal, crepuscular circadian pattern in Apennine bears. Our results revealed that under the historical and current conditions of human disturbance, such as forestry, livestock husbandry, tourism, outdoor activities and, in the external buffer area of the park, hunting and increased resource use by humans^39^, Apennine brown bears were not forced to adopt a marked nocturnality. By means of telemetry, higher activity during crepuscular periods was recorded for other European brown bears populations as well (Serbia:^27^; Sweden:^22,36^; Slovenia and Croatia:^29^). Activity during crepuscular and nocturnal periods was estimated for bears in northern Italy^28^ and in the Pyrenees^30^ as well, even though these have been established through camera trapping, therefore yielding results not necessarily comparable to ours^58^.

A crucial factor influencing activity rhythms in brown bears is the timing of feeding in relation to the availability of resources across the diel period^23,59^. As bears mainly detect fruits and berries through sight^26^, the bimodal activity pattern exhibited by Apennine bears and other European populations could be considered a trade-off between an optimal foraging strategy and the risk of encountering man. In southcentral Sweden, during summer and before the start of the bear hunting season, the extent of diurnal activity in adult bears was negatively influenced by road density^60^. Therefore, bear populations that live in relatively small areas with high road and human density, such as the Apennine brown bears in the PNALM, may be expected to adopt nocturnal circadian patterns to temporally displace from human activity; yet, the conditions found in our study area and, perhaps most importantly, the longtime protected status of the bear population^61,62^, are such that, while clearly avoiding diurnal activity patters, Apennine bears are not forced to adopt a markedly nocturnal circadian rhythm.

We found no differences in daily activity between male and female bears residing in the PNALM, disproving our H2 and in line with previous studies^3,60^. Yet, it is noteworthy that, even though its coefficient is not significant, our best selected model contains sex as a factor, both in interaction with season and with the hour of the day. We therefore suspect that such an effect might have a role in our bear poulation, but the limited number of individuals in our study, coupled with a high inter-individual variability, might correspond to a low statistical power. Furthermore, our sample of Apennine female bears did not include females with cubs that, to reduce the risk of infanticide especially during the mating season^63^, may exhibit higher diurnal and lower nocturnal activity to decrease the chances of encounter with adult males^27,36^.

We expectedly found bears to be more active in early summer, coincident with the mating period, confirming our P1. Bears displayed more pronounced peaks of activity during the mating season compared to the other seasons^63^. This behavior has been reported also in other European brown bear populations, such as in Greece^64^, and in Serbia^27^, where adult males and females exhibit higher movement rates and synchronized peaks of activity as they roam to find a partner. On the other hand, bears’ circadian rhythms during the rest of the annual active period could be interpreted as a baseline activity pattern that bears follow outside the mating season. Accordingly, our results suggest that, during the years of our study, the resources used by bears were sufficiently available so that they did not need to increase their activity levels during any particular season to meet their energetic requirements^26,65^. However, given our small sample size we could not evaluate a possible effect of the year, although it is reasonable to expect important variations from year to year as a function of fluctuations in quality and abundance of key foods^43^. As such, a larger sample size over a longer number of years may provide the opportunity to further evaluate this effect.

In the human-modified landscape of the PNALM, contrary to our P2, we failed to detect increased nocturnality of bears during summer, when human presence and activity in the park increase^66^. Switching to a greater nocturnality during summer could translate into less efficient foraging, as in this period bears increase consumption of berries and other fleshy fruits^44^, which are located and selected mainly by sight^26,36^. Bears in Sweden increase their diurnal activity during their hyperphagic period (i.e., summer − early fall), when they mainly feed on berries, although this modification is less evident in areas with higher intensity of human disturbance^36^. Indeed, during summer months female bears in central Italy have been recorded to avoid roads and to select high tree density structures for sheltering throughout daylight hours within the home range scale^61^. As such, bears may be adopting habitat-mediated strategies so that they do not need to adopt nocturnal patterns in order to avoid the higher human pressure^33,35,67^. Furthermore, extensive use of beech forests at higher altitudes^61,62^, which are highly efficient in temperature regulation^68^, may allow Apennine bears to reduce heat stress during summer, and therefore to maintain their activity rhythms also during this period^24^.

Although we obtained mixed results concerning response by Apennine bears when moving closer to anthropic features of the landscape, during various seasons we detected an increase in their movement rate when they approached infrastructures and human settlements, generally confirming our P3. In spring, bears increased their movement rates in proximity to both primary and secondary roads, while in late summer bears only marginally increased their pace in proximity to secondary roads. It is likely that the higher movement rates of bears when they approach infrastructures is a response to a more intense perception of risk, similarly to what was reported for bears in Greece^64^. As a matter of fact, vehicle collisions with bears in the PNALM has been a leading cause of mortality^39,62^. Alternatively, moving close to linear infrastructures may allow bears to travel faster across the landscape^69^. During fall and partly during late summer, however, bears do not exhibit a significant response in terms of movement rate when approaching roads. This could be indicative of food availability in these areas during the hyperphagic period^61,70^, indicating a trade-off between enhanced foraging opportunities and risk prevention. In the Apennines, secondary roads have been associated to sink-like habitats for bears at the landscape scale^62^, and the non-significant change in movement rate that we revealed in their proximity during the hyperphagic period possibly indicates a responsible mechanism. In addition, the greater activity of bears that we detected during the mating season, likely due to the intense search for a partner^63^, may be the reason behind the apparent lack of any risk response towards infrastructures that bears displayed during early summer. Similarly, movement rates of bears when approaching human settlements increased in spring and late summer, while their response was non-significant during early summer and fall. Although foods such as livestock, bees, and domestic fruits are included in the Apennine bears’ diet, no marked dependency on anthropogenic resources was found in this bear population^43,44^. Yet, it is possible that proximity to settlements affects bears’ movement rates through enhanced food availability^61^, again suggesting a trade-off between foraging opportunities and risk avoidance. Fine-scale habitat modeling in relation to location of key foods used by bears is needed to verify this hypothesis. Not only the presence of food items near roads and settlements could represent sink-like habitat features^61,62^, but they could also favor food-conditioning and habituation in bears, that may ultimately result in problematic behavior and conflict with humans^71^.

Using a Bayesian modeling framework enabled us to explore a previously unknown aspect of the ecology of the endangered Apennine bear population using a realistic, cyclical representation of circadian rhythms with a precision of results expectedly higher compared to more classical, frequentist approaches^22,36,54^. Beyond our own study population, this study enhances knowledge of bears’ behavior in highly human-modified landscapes, a condition that more bear populations will face in the near future both in Europe and elsewhere^15^. Yet, whereas we were able to identify a clear pattern at the population level, individual differences in circadian activity (i.e., as measured by ρ^2^) represent an important source of variability, as expected from previous studies on activity patterns of brown bears^60^. Additionally, the temporal resolution of our relocations (1 location/h), and the fact that movement rates do not fully represent all the complete activity carried out by bears (e.g., stationary actions such as grooming or feeding on localized resources), inherently limit the behavioral resolution of our findings. From a demographic perspective, we suggest that further research on the activity of this imperiled bear population should include younger age classes and female with cubs, as both are important for the recovery of this population^47^.

European bears have been consistently reported to exhibit crepuscular circadian patterns, with varying levels of nocturnal activity mainly linked to variations in age class^29,60^, impact by hunting^34^, and time of the year^27,60^. Instead, brown bears in North America exhibit a variety of activity rhythms, from nocturnal, to crepuscular, to diurnal, being highly dependent on the time of the year and on the level of anthropization of the landscape^25–27^. As such, brown bears in Europe may not display the same flexibility in circadian activity patterns as bears in North America, as their long history of persecution and the high level of anthropization of the landscape may have induced enduring physiological modifications in terms of circadian rhythms^31,32^. The essentially crepuscular pattern of activity displayed by bear populations in Europe could be both the result of a long process of coevolution with humans^32^, and the result of a more immediate process, such as a learned strategy transmitted by cultural inheritance^29,60^. Regardless, the possible fitness effects of human-induced modifications in the activity patterns of large carnivores, and specifically bears, are still unknown and represent an open research topic.

## Supplementary Information

Supplementary Information.

## Data Availability

The data presented in this study are available on request from the corresponding author. The data are not publicly available due to privacy restrictions.

## Code availability

The code of the best selected model is available at: https://github.com/auroradonatelli/circadian-models.
