# Supplementary material for: Circadian activity of small brown bear populations living in human-dominated landscapes
Source: Sci Rep. 2022 Sep 22;12:15804. doi: 10.1038/s41598-022-20163-1 (PMC9499929; doi:10.1038/s41598-022-20163-1)

Circadian activity of small brown bear populations living in human-dominated landscapes

Aurora Donatelli[1], Gianluca Mastrantonio[2], Paolo Ciucci[1]*

[1] Department of Biology and Biotechnologies "Charles Darwin", Sapienza University of Rome, Rome, Italy.

[2] Department of Mathematics (DISMA), Politecnico di Torino, Torino, Italy.

* Corresponding author: paolo.ciucci@uniroma1.it

Supplementary Information

Figure S1 – Pair-wise differences between the circadian rhythms of female and male Apennine bears (i.e., females' mean predicted values– males' mean predicted values), based on 26,880 GPS locations of 11 female and 7 male bears resident in the Abruzzo, Lazio and Molise National Park (central Italy, 2005−2010). (a) Mean hourly activity of female bears relative to male bears in spring; (b) mean hourly activity of female bears relative to male bears in early summer; (c) mean hourly activity of female bears relative to male bears in late summer; (d) mean hourly activity of female bears relative to male bears in fall. Dashed lines represent 95% credibility intervals.

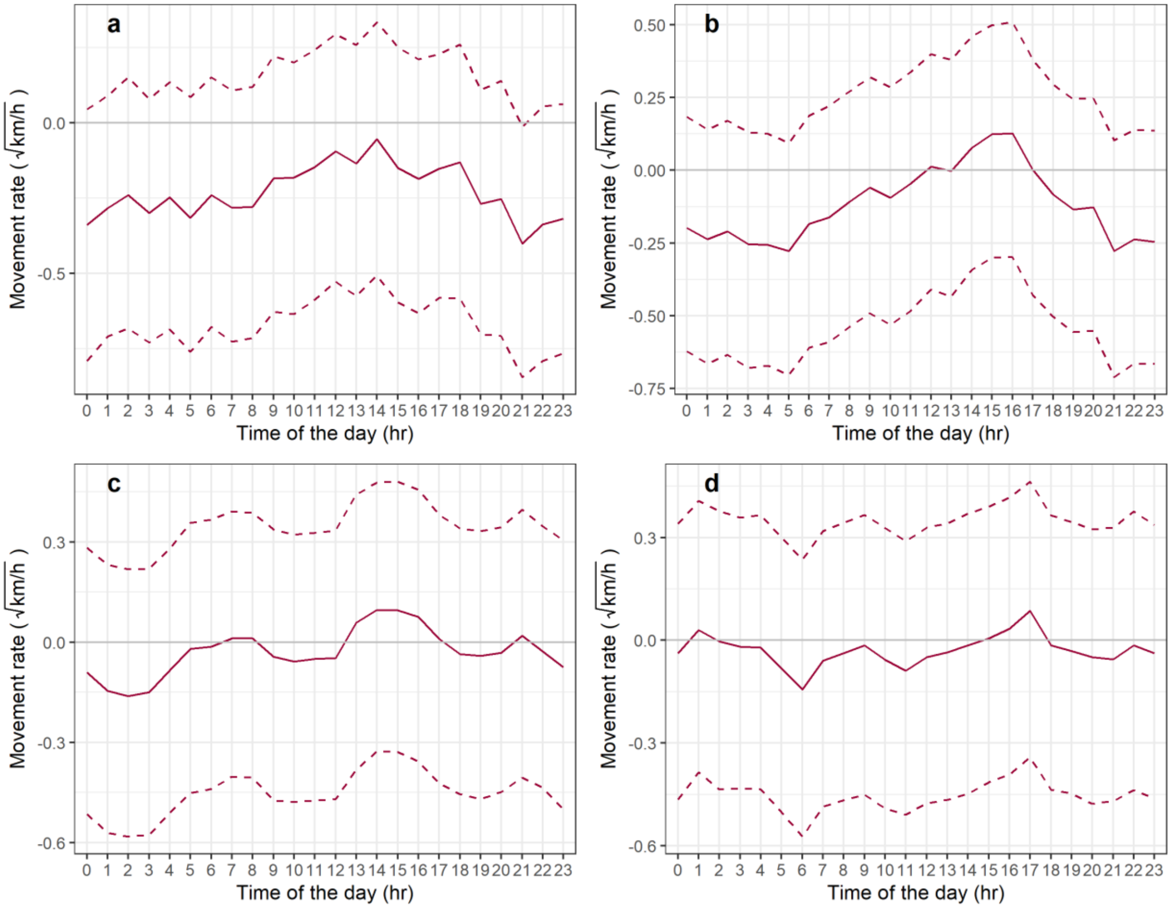

Figure S2 – Pair-wise differences between seasonal circadian rhythms of Apennine bears, based on GPS locations of 11 female and 7 male bears resident in the Abruzzo Lazio and Molise National Park (central Italy, 2005−2010). (a) Mean hourly activity in spring relative to early summer (activity rhythm in spring – activity rhythm in early summer); (b) mean hourly activity in spring relative to late summer (activity rhythm in spring – activity rhythm in late summer); (c) mean hourly activity in spring relative to fall (activity rhythm in spring – activity rhythm in fall); (d) mean hourly activity in early summer relative to late summer (activity rhythm in early summer – activity rhythm in late summer); (e) mean hourly activity in early summer relative to fall (activity rhythm in early summer – activity rhythm in fall); (f) mean hourly activity in late summer relative to fall (activity rhythm in late summer – activity rhythm in fall). Dashed lines represent 95% credibility intervals.

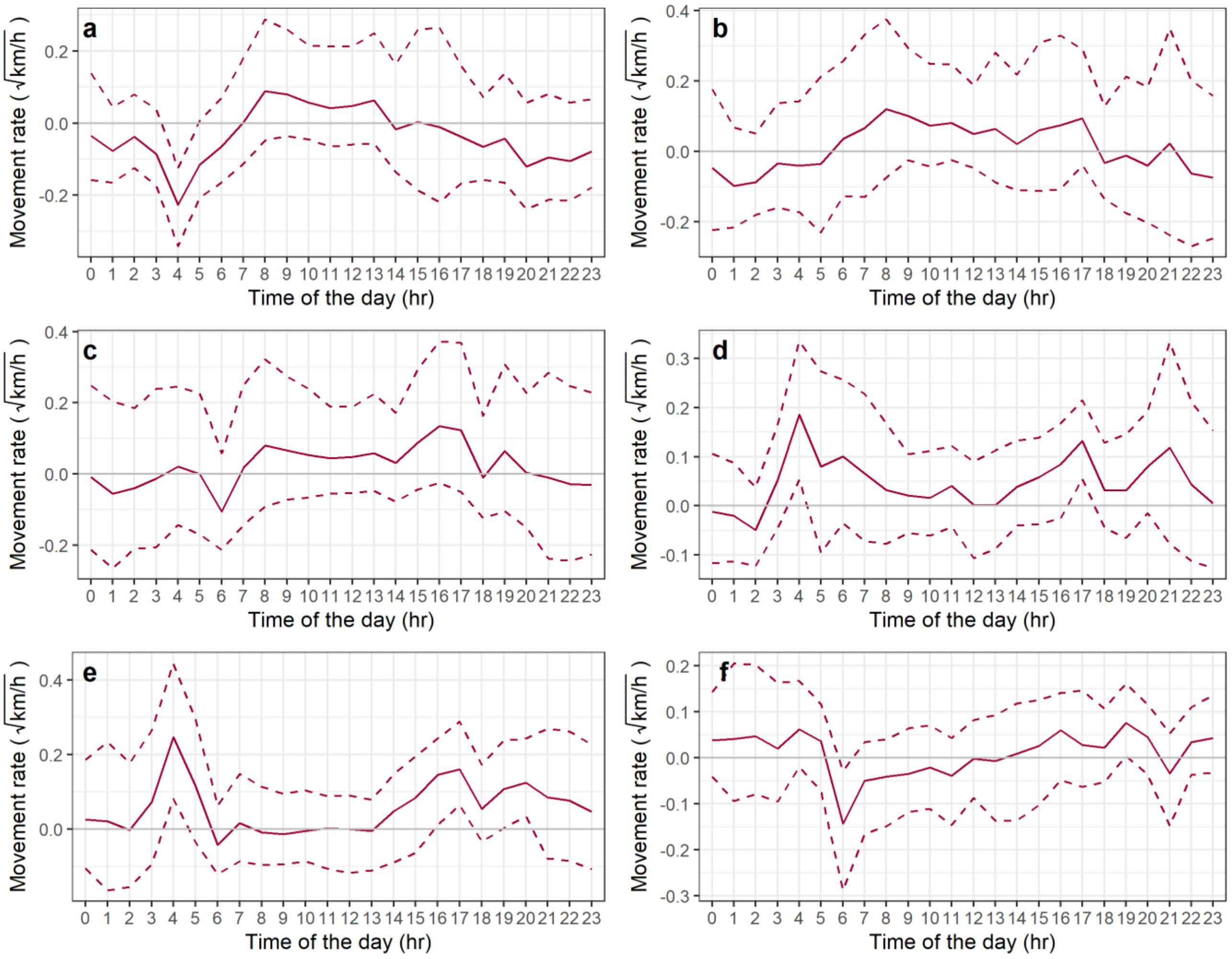

Figure S3 – Effect of the distance to primary roads in spring on the movement rate of Apennine bears, based on a sample of 26,880 fixes from 18 adult individuals. Dashed lines represent 95% credibility intervals.

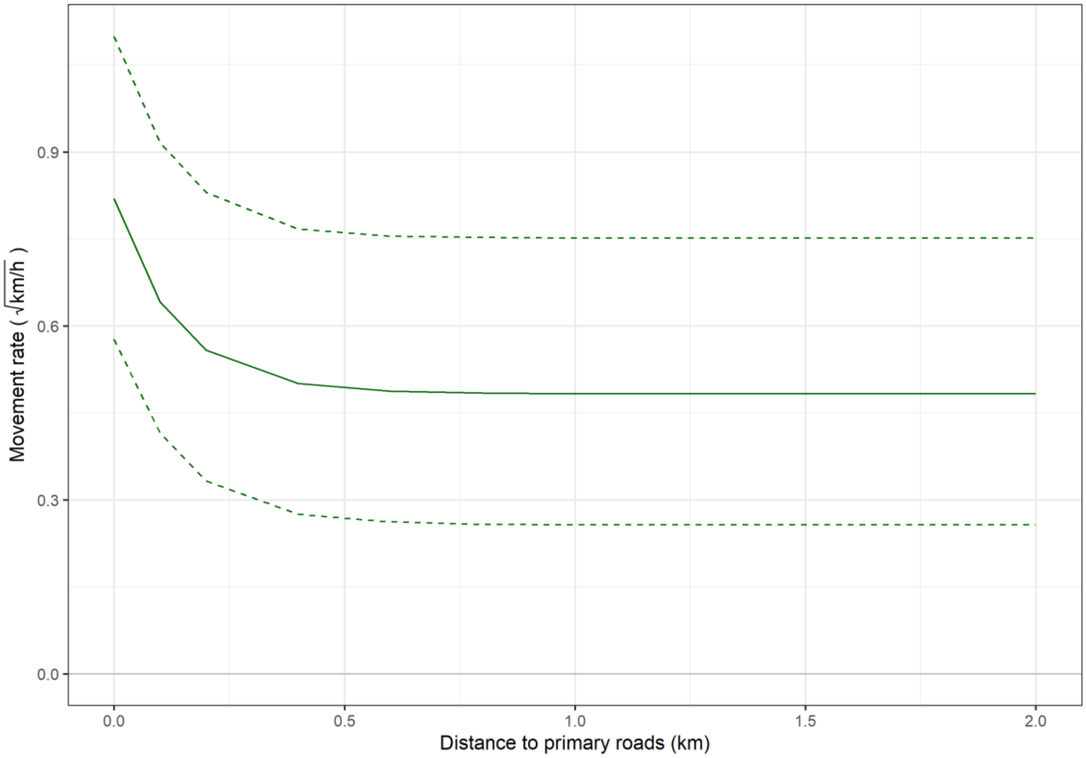

Figure S4 – Effect of the distance to secondary roads in spring (a) and late summer (b) on the movement rate of Apennine bears, based on a sample of 26,880 fixes from 18 adult individuals. Dashed lines represent 95% credibility intervals.

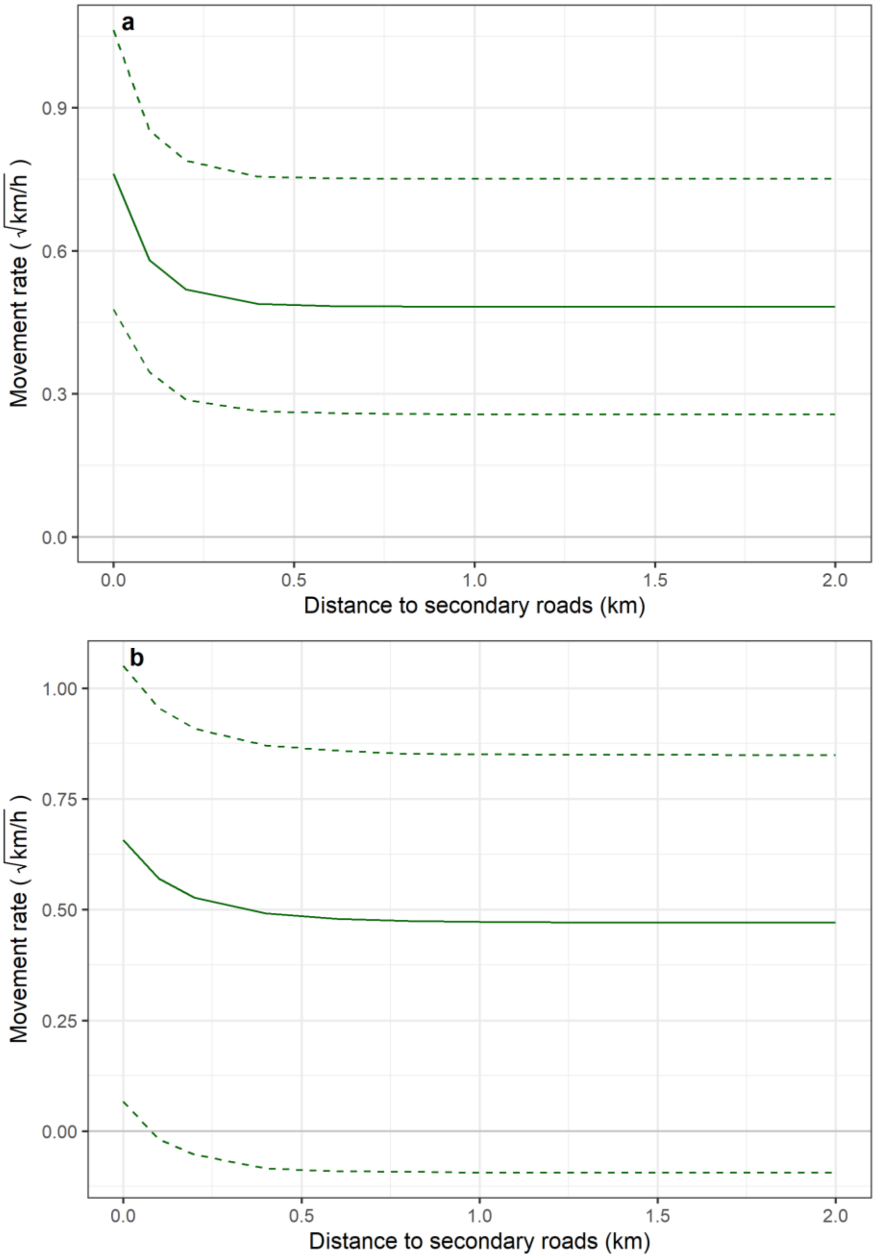

Figure S5 – Effect of the distance to human settlements on the movement rate of Apennine bears, based on a sample of 26,880 fixes from 18 adult individuals in spring (a) and late summer (b). Dashed lines represent 95% credibility intervals.

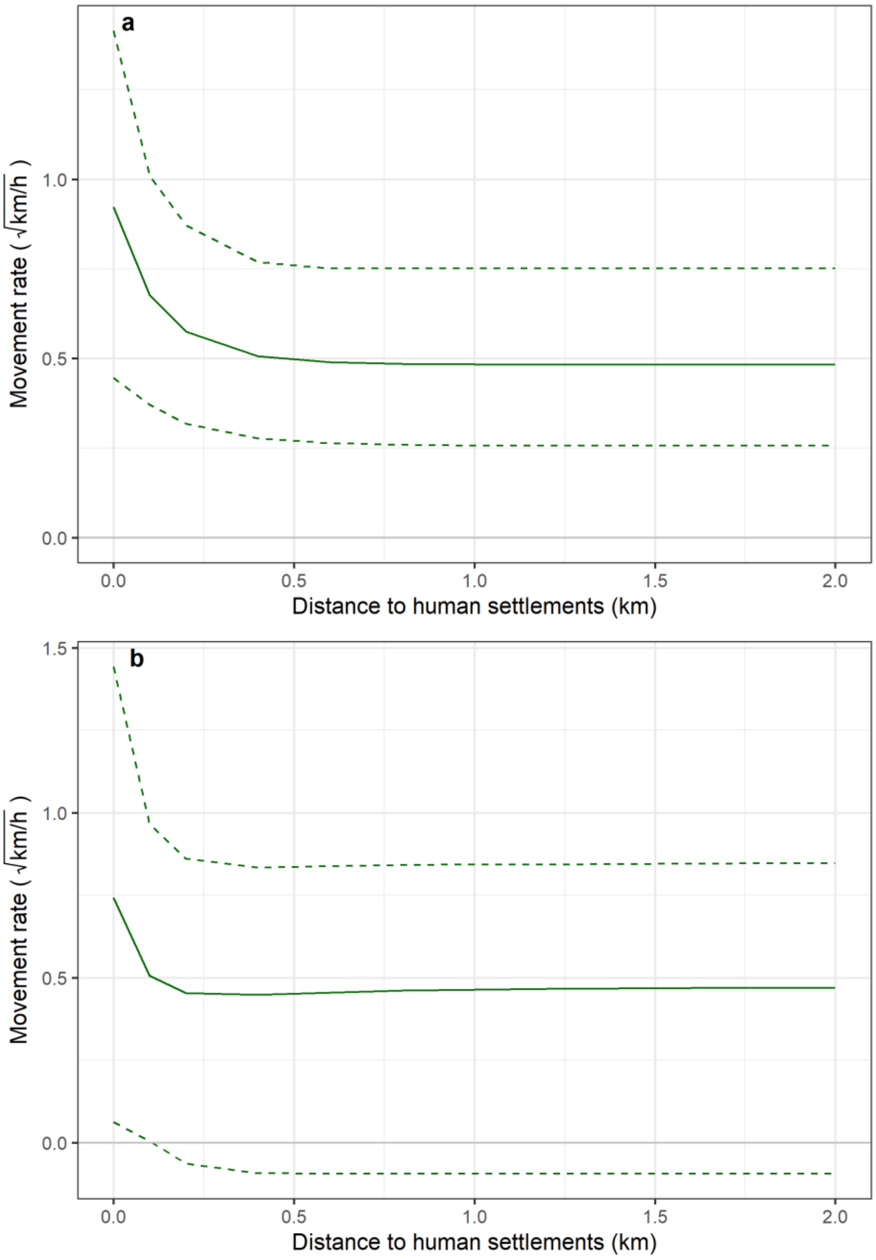

Figure S6 – Boxplots of distances to primary roads in relation to time of the day of 18 adult individuals of Apennine bears, based on a sample of 26,880 fixes, in spring (a), early summer (b), late summer (c) and fall (d). The extremes of the boxes represent the first and the third quartiles, and the middle bold line is the median, while the whiskers extend to 1.5 times the interquartile range above the upper quartile and below the lower quartile.

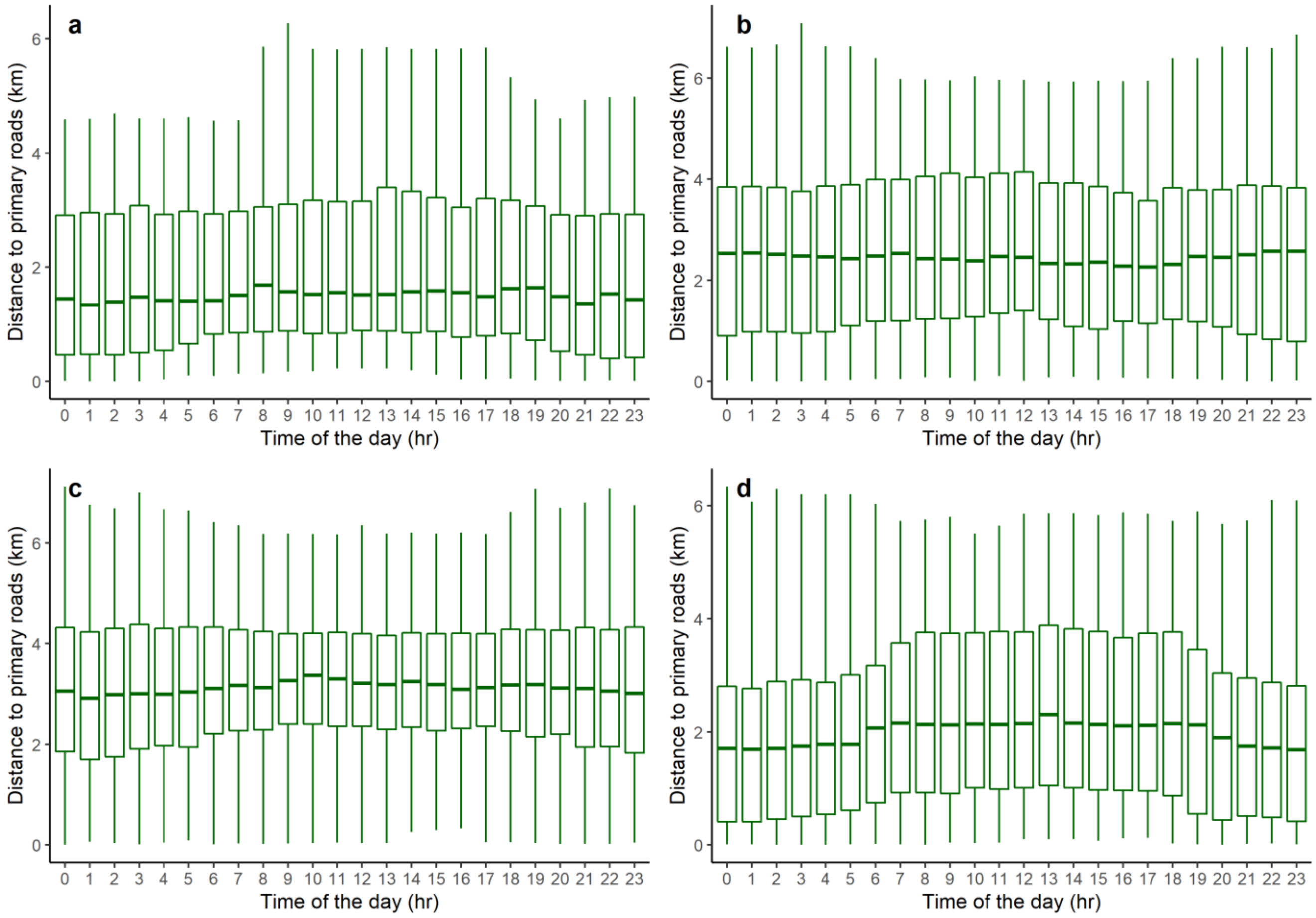

Figure S7 – Boxplots of distances to secondary roads in relation to time of the day of 18 adult individuals of Apennine bears, based on a sample of 26,880 fixes, in spring (a), early summer (b), late summer (c) and fall (d). The extremes of the boxes represent the first and the third quartiles, and the middle bold line is the median, while the whiskers extend to 1.5 times the interquartile range above the upper quartile and below the lower quartile.

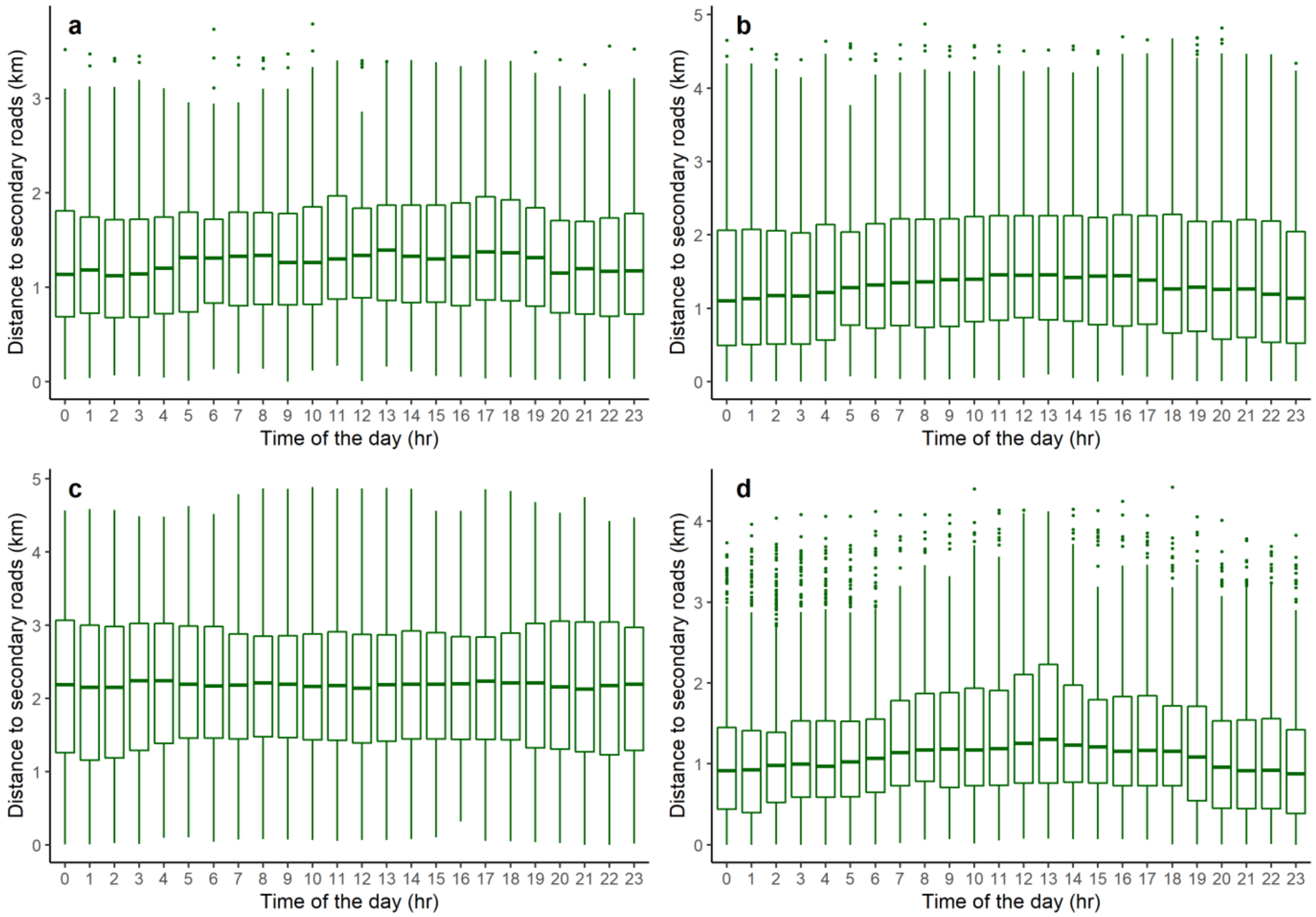

Figure S8 – Boxplots of distances to human settlements in relation to time of the day of 18 adult individuals of Apennine bears, based on a sample of 26,880 fixes, in spring (a), early summer (b), late summer (c) and fall (d). The extremes of the boxes represent the first and the third quartiles, and the middle bold line is the median, while the whiskers extend to 1.5 times the interquartile range above the upper quartile and below the lower quartile.

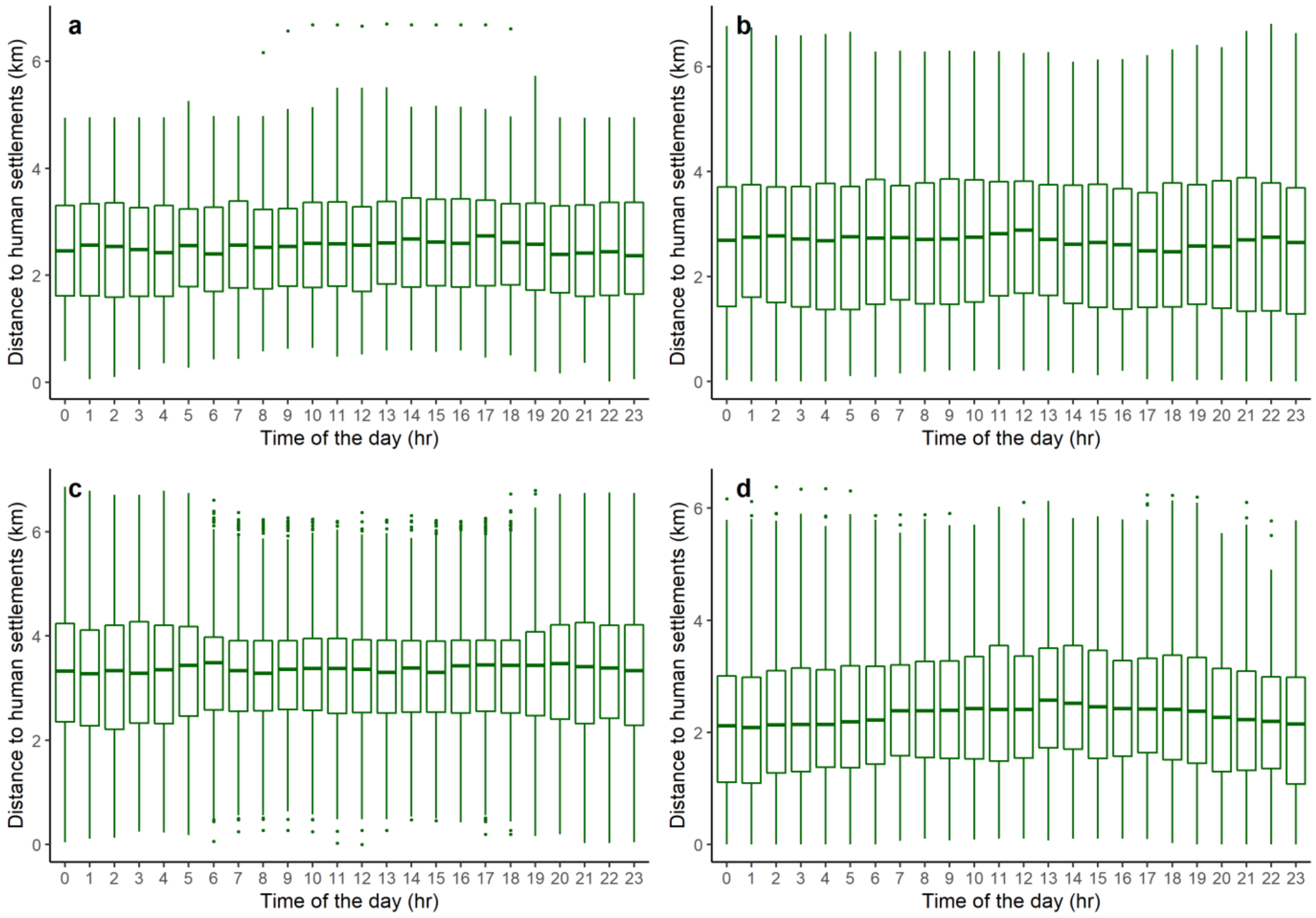

Supplement: Supplementary file 1 — Supplementary Information. [file 41598_2022_20163_MOESM1_ESM.pdf]
